# The prevalence of pain and disability one year post fracture of the distal radius in a UK population: A cross sectional survey

**DOI:** 10.1186/1471-2474-9-129

**Published:** 2008-09-29

**Authors:** Catherine M Moore, Jo Leonardi-Bee

**Affiliations:** 1 Division of Physiotherapy Education, University of Nottingham, Nottingham, UK; 2 Division of Epidemiology and Public Health, University of Nottingham, Nottingham, UK

## Abstract

**Background:**

A fracture of the distal radius is a commonly occurring fracture and accounts for a third of all fractures in the elderly. Thus far, one year estimates of pain and disability following a fracture of the distal radius have been reported on Canadian populations. The primary aim of this study is to investigate the prevalence of pain and disability in a UK population one year post fracture of the distal radius.

**Methods:**

A cross-sectional survey was undertaken, of all subjects suffering a fracture of the distal radius between October 2005 and February 2006 in Nottingham, UK. Primary outcomes used were the VAS for pain and the DASH for disability. Prevalence of pain and disability were calculated and odds ratios presented for associations between demographics, pain and disability.

**Results:**

93/264 (35%) subjects responded to the questionnaire. 6 subjects did not fulfill the inclusion criteria and were excluded from further analysis. 11% of subjects reported moderate to very severe pain. 16% of subjects reported moderate to very severe disability. Statistically significant associations were found between pain medication usage for the wrist fracture and moderate to very severe pain (OR 11.20, 95% CI 2.05 – 61.23). Moderate to very severe disability was associated with older age (OR 6.53, 95%CI 1.65 – 25.90) and pain medication usage for the wrist fracture (OR 4.75, 95% CI 1.38 – 16.37). Working was associated with a reduction in risk of moderate to very severe disability (OR 0.14, 95% CI 0.03 – 0.67).

**Conclusion:**

This study demonstrates that there are a small proportion of patients who are still suffering moderate to very severe pain and disability one year post fracture of the distal radius. The study also demonstrates that there are significant associations between characteristics of the patients and the level of pain and disability. This highlights the need for further research into the most appropriate management of these patients in order to reduce this burden of pain and disability, particularly as this is a predominantly elderly patient group.

## Background

Fractures are one of the most common reasons for attendance at the Accident and Emergency (A & E) department of any hospital, making it a very expensive aspect of any health care system [1]. Fracture rates in the UK have been estimated at 10 per 1000 person years for males and 8 per 1000 person years for females [2]. Fracture rates are similar for the US being estimated at approximately 8.47 per 1000 person years for both males and females, with adolescents being the most frequent sufferers [1]. Below the age of 55 fractures are more common in males whereas over 55 years this trend gradually reverses [2] due to factors such as osteoporosis [2,3].

A fracture of the distal radius (FDR) is an injury which occurs predominantly in older females and is usually caused by a fall onto an outstretched hand [4]. An annual incidence rate, for FDR, of 36.8/10,000 person years in women and 9.0/10000 person years in men has been estimated [5]. This type of fracture involves an injury to the distal end of the radial bone which forms part of the wrist joint. It is a fracture which occurs more frequently than any other wrist fracture, posing an extreme strain on health services [6].

Following any injury a certain level of pain can be anticipated and the FDR is no exception. Chronic pain can be described as pain which persists beyond the point at which the tissues would be expected to heal [7]. No general estimates of the number of people who experience chronic pain after a fracture can be found; this is probably as fractures differ in severity and impact from bone to bone. However, it has been estimated that chronic pain after a FDR could affect as much as 30% of patients, with 11% of patients reporting moderate to very severe pain after one year [8]. These estimates are based on a Canadian cohort study, and until now estimates have not been available for the UK.

Disability is another consequence of a FDR, particularly as it largely affects the elderly population. The disability, resulting from a FDR, may be the inability to perform activities of daily living such as dressing or cooking, meaning the difference between independent living and the demand for social care. This creates an even greater economic strain on the individual and society than just the burden of treatment for the fracture alone. Canadian estimates show that 16% of patients, following a FDR, will experience some form of disability making this a huge public health burden [8]. However, no estimates for the UK can be found. This is the first study to focus on the prevalence of pain and disability in a UK population one year following a FDR.

## Methods

A cross sectional postal survey, using a battery of standardised questionnaires, was targeted at any patient who suffered a FDR between 5th October 2005 and 28th February 2006, in the Nottingham district of the UK. Potential research subjects (n = 268) were identified by a search through the computerised medical documentation kept at the University Hospital Nottingham, Queens Medical Centre campus (QMC). Patients included were those adults who had experienced a FDR which was diagnosed via x-ray on admission to the A and E department, one year before the time of the study. Patients were excluded if they had been admitted for multiple fractures of the upper limb at the time of injury and if they were below the age of 18 years at the time of the study.

264 subjects met the inclusion criteria of the study and an invitation questionnaire was posted to them with a covering letter; patient information sheet and consent form inviting them to take part in the study. If they wished to take part, they were asked to complete the questionnaire and consent form and return them both, in a pre-stamped addressed envelope provided. Recruitment was encouraged by sending a reminder letter to patients two weeks after the invitation letter. On receipt of the completed questionnaire and consent form, the patients' medical records were obtained. These were scrutinised to identify the type of fracture they had experienced and what management they had received.

### Outcome measures

The battery of questionnaires requested demographic information including information on present medication usage for the wrist injury and the main outcome measures of the Disability of the Arm Shoulder and Hand (DASH; see additional file 1) and Visual Analogue Scale (VAS; see additional file 2). The demographic information included age, sex, hand dominance, injured hand, ongoing compensation, occupation, work status, smoking history, ethnic origin and previous injury to the same arm. The DASH is a validated 30 item questionnaire that evaluates disability of the upper limb with a five likert-like response option for each item of the questionnaire [9]. The VAS is a 0 – 10 numerical validated scale which the person rates their pain out of a maximum of 10 (10 meaning the greatest level of pain imaginable to the individual) [10].

### Analysis

Analysis was performed using SPSS (version 14.0). For the continuous variables (DASH and VAS scores), non normal distributions were seen, therefore, the data were summarised using the non parametric descriptors median and Inter-quartile ranges (IQR).

The raw VAS data gave a score out of 10 for each patient. In order to make this score more meaningful the raw VAS score was converted to ordinal data following the methodology described by MacDermid et al (2003) [8]. Once the VAS scores had been converted to the categorical groups of – no pain (0/10), minimal pain (1–2/10), mild pain (3–4/10), moderate pain (5 – 6/10), severe pain (7 – 8/10), very severe pain (9 – 10/10) the prevalence of pain was calculated.

The raw DASH scores gave a score out of 100 for each patient; this was the percentage disability score and were converted to categorical groups of – no disability (0%), minimal disability (1 – 20%), mild disability (21 – 40%), moderate disability (41 – 60%), severe disability (61 – 80%), very severe disability (81% – 100%) similarly following the methodology described by MacDermid et al (2003) [8]. The prevalence of disability was then calculated.

### Associations between demographics and pain/disability

Associations between binary data were assessed using chi-square tests or Fisher exact test if small expected frequencies were seen. Results are presented as odds ratios (OR) with 95% confidence intervals. A multivariate logistic regression analysis was performed on those variables identified as statistically significant from the univariate associations. Statistical significance was set at p < 0.05. Descriptive statistics were calculated for numbers of respondents and demographic profile of the sample.

### Margin of error/sample size

A sample of 80 people were required to obtain a 95% confidence interval of +/- 10% around a prevalence estimate of 30% [8]. To allow for an expected 30% response rate to the questionnaire, the sample size was approximately tripled and a total of 264 questionnaires were delivered.

### Ethical approval

Ethical approval for the study was granted by the Local Research Ethics Committee (ref 06/Q2402/64) and the Research and Development Department (R&D) at the Nottingham University Hospitals Trust (NUH) (ref 06AE003).

## Results

### Sample

Computerised records identified 268 patients, of which 4 had died, thus a total of 264 questionnaires were posted to the remaining subjects. Of the 264 questionnaires posted 93 (35%) subjects returned completed questionnaires that could be used in the analysis. 40 (15%) subjects returned the cut off reply slip requesting no further contact, 16 (6%) were not known at the address that was available from the records at the QMC, 2 (2%) reported never having experienced a fracture and 112 (42%) made no response to the questionnaire at all.

### Responders demographics

Table 1 shows the main characteristics of the responders (n = 93). The mean age of the responders was 58 (SD 18) with an age range of 22 to 89 years. 81% of this group were female, 90% were right hand dominant, 45% had injured their right hand, half of the group (50%) had injured their dominant hand and 7% were pursuing a compensation claim. 13% reported that they were current smokers, 32% were ex smokers and 55% had never smoked. The respondents were more likely to be older (p = 0.017) and female (p < 0.008).

**Table 1:** Characteristics of the 93 responders

Mean age (SD)	58 (18); range 22 – 89	% (n=)
Sex	Female	81% (76)
Dominance	Right	90% (84)
Injured Hand	Right	45% (42)
	Left	55% (51)
Dominant Hand Injured	Yes	50% (46)
Undergoing compensation	Yes	7% (6)
Diagnosis in Medical Documentation	Un-displaced Radial Fracture	28% (25)
	Un-displaced Radial and Ulna fracture	2% (2)
	Displaced Radius	32% (29)
	Displaced Radius and Ulna	30% (27)
	Other fracture	3% (3)
	No fracture	3% (3)
		4 missing
Intra/extra-articular fracture	Intra-articular	35% (30)
	Extra-articular	9% (8)
	Unknown	56% (47)
		8 missing
Management of Fracture	Open reduction internal fixation	21% (19)
	Closed reduction and cast	41% (37)
	Cast alone	32% (28)
	Other	6% (5)
		4 missing
Working	Yes	53% (49)
Not Working Due to Injury	Yes	1% (1)
Occupation	Professional	17% (15)
	Supervisory	14% (12)
	Skilled manual	13% (11)
	Unskilled manual	14% (12)
	Retired	43% (38)
		5 missing
Pain Medication Usage	Yes	23% (21)
Medication Frequency	Once a Month	20% (5% of full sample) (4)
(% of the responders	Once a Week	20% (5% of full sample) (4)
who did Require medication)	Once a Day	25% (6% of full sample) (5)
	Twice a Day	20% (5% of full sample) (4)
	More often	15% (3% of full sample) (3)
		1 missing
Previous Injury to Same Arm	Yes	18% (17)
Smoking status	Current Smoker	13% (12)
	Ex smoker	32% (30)
	Non smoker	55% (51)
Ethnic Origin	White British	96% (83)
	Asian Indian	2% (2)
	Asian Pakistani	1% (1)
	Other	1% (1)
		6 missing
Rehabilitation	Physiotherapy	56% (50)
	Occupational Therapy	11% (9)
	Both services	9% (8)

SD = Standard deviation

The diagnosis available in the medical records showed that the most frequently occurring injury was a displaced fracture of the distal radius (32%). 30% of patients suffered a displaced fracture of the distal radius and ulna, 28% suffered an un-displaced fracture of the distal radius and 2% suffered an un-displaced fracture of both the distal radius and ulna. 6% of patients had been misdiagnosed on admission to A & E. These patients had been categorised as a FDR when they had actually experienced another type of injury (3% different fracture type, 3% no fracture). These patients were removed from the data set leaving 87 patients for further analysis.

The available information on classification of fracture type showed that 35% were classified as an intra-articular fracture, 9% as an extra-articular fracture and 56% were not classified in the medical documentation. The management of FDR was predominantly via a closed reduction of the displaced fracture and plaster cast (41%), 32% were managed by plaster cast alone and a further 21% underwent an open reduction and internal fixation. 6% of respondents had not suffered a fracture of the distal radius and were classified as other.

A little over half (53%) were working and only 1% were not working due to their injury. 43% of the subjects were retired, 23% reported the need to use pain medication for the injury, 18% had had a previous injury to the same arm, 96% of the sample were White British, 56% had received physiotherapy and 11% had received OT for their injury. 9% of the sample had received both physiotherapy and OT and 41% received no rehabilitation services at all.

### Prevalence of pain

**Figure 1:**
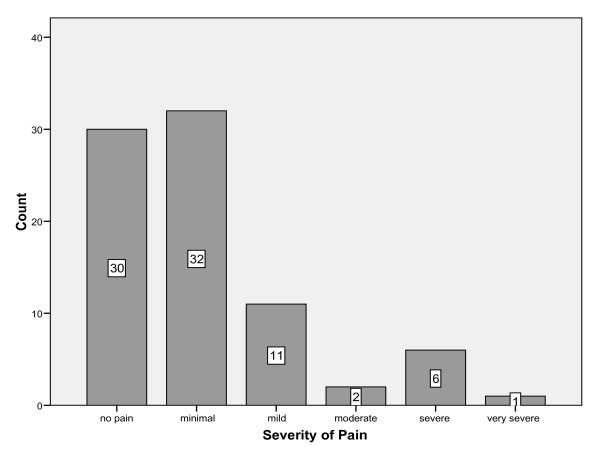
Prevalence of pain in responders (5 missing).

The median VAS score was 1 with an inter-quartile range of 0 to 2.5, however, these scores as raw data are meaningless and, therefore, categories of pain severity were created. 37% of the patients had no pain, 39% had minimal pain, 13% had mild pain, 2% had moderate pain, 7% had severe pain and 1% reported very severe pain (figure 1). Hence 63% of the subjects had some degree of pain one year post fracture and 11% had moderate to very severe pain. Similar distributions were seen for males and females.

### Prevalence of disability

**Figure 2:**
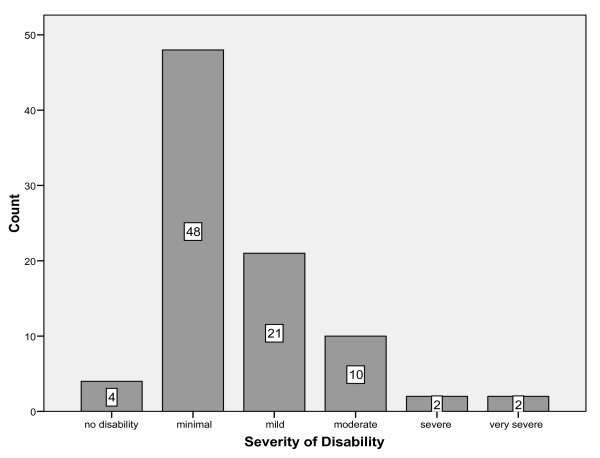
Prevalence of disability in responders (0 missing).

The median DASH score was 14 with an inter-quartile range of 7 to 28 these were converted to categories of severity. 95% of patients had some degree of disability with 16% of people being left with a degree of disability (moderate, severe and very severe) that would severely interfere with their life (figure 2). Similar distributions were seen between males and females.

### Univariate and multivariate analyses

Results from the univariate analysis showed that patients over 65 were almost 7 times (OR 6.53, 95% CI 1.65 – 25.90, p = 0.003) more likely to be moderately to very severely disabled than their younger counterparts (Table 2). Additionally those with moderate to very severe disability were 86% less likely (OR 0.14, 95% CI 0.03 – 0.67, p = 0.006) to be working and nearly 5 times (OR 4.75, 95% CI 1.38 – 16.37, p = 0.015) more likely to require pain medication. However, in a multivariate analysis of the significant variables only pain medication usage remained statistically significant (p = 0.008) (table 3). Significant associations for pain were only seen with pain medication usage (OR 11.20, 95% CI 2.05 – 61.23, p = 0.004).

**Table 2:** Associations between responder characteristics, pain and disability

		Disability (DASH)			Pain (VAS)		
Characteristic		OR†	95% CI	P value	OR ‡	95% CI	P value

Age	65+	6.53	1.65 – 25.90	0.003*	1.64	0.38 – 7.10	0.71#
	17 – 64	1.00 (ref)			1.00 (ref)		
Sex	Female	2.80	0.37 – 23.36	0.45#	0.29	0.06 – 1.40	0.13#
	Male	1.00(ref)			1.00 (ref)		
Injured hand	Left	0.77	0.23 – 2.60	0.68	1.39	0.32 – 5.98	0.72#
	Right	1.00 (ref)			1.00(ref)		
Dominant hand injured	No	0.63	0.19 – 2.09	0.44	1.11	0.26 – 4.79	1.00#
	Yes	1.00 (ref)			1.00 (ref)		
Undergoing compensation	No	~	~	~	3.38	0.31 – 37.00	0.34#
	Yes	1.00 (ref)			1.00 (ref)		
Diagnosis from medical	displaced	**~**	**~**	**~**	0.89	0.15 – 5.27	1.00#
	Un-displaced	1.00 (ref)			1.00 (ref)		
Management of fracture	Open	1.45	0.34 – 6.17	0.70#	0.59	0.07 – 5.28	1.00#
	Closed	1.00 (ref)			1.00 (ref)		
Working	Yes	0.14	0.03 – 0.67	0.006*	0.90	0.21 – 3.86	1.00#
	No	1.00 (ref)			1.00 (ref)		
Using pain medication	Yes	4.75	1.38 – 16.37	0.015#*	11.20	2.05 – 61.23	0.004#*
	No	1.00 (ref)			1.00 (ref)		
Previous injury	No	0.78	0.16 – 3.92	1.00#	0.67	0.08 – 5.92	1.00#
	Yes	1.00 (ref)			1.00 (ref)		
Smoking status	Smoker	1.47	0.16 – 13.57	1.00#	~	~	~
	Ex smoker	0.56	0.16 – 1.94	0.52#	0.31	0.07 – 1.41	0.14#
	Non smoker	1.00 (ref)			1.00 (ref)		
Rehabilitation	PT alone	1.78	0.44 – 7.27	0.49#	0.92	0.14 – 5.92	1.00#
	OT alone	~	~	~	~	~	~
	Both	1.35	0.13 – 13.47	1.00#	0.54	0.04 – 6.84	0.54#
	Neither	1.00 (ref)			1.00 (ref)		

† OR is the odds ratio for moderate to very severe versus non to mild disability

CI Confidence interval

‡ OR is the odds ratio for moderate to very severe pain versus non to mild pain

# Fisher's exact test utilized

~ Unable to calculate OR due to zero cells

* Significant P value

**Table 3:** Results of the multivariate analysis

		Disability (DASH)			Pain (VAS)		
Characteristic		OR	95% CI	P value	OR±	95% CI	P value

Age	65+	1.11	0.46 – 19.89	0.247			
	
	17 – 64	1.00 (ref)					

Working	Yes	-1.48	0.27 – 1.95	0.177			
	
	No	1.00 (ref)					

Using medication	Yes	1.84	1.53 – 25.74	0.008*	11.20	2.05 – 61.23	0.004*
	
	No	1.00 (ref)			1.00		

OR is the odds ratio for moderate to very severe versus non to mild disability

OR‡ is the odds ratio for moderate to very severe pain versus non to mild pain

CI Confidence interval

* Statistically significant at 5% level

## Discussion

The results of the present study show that 63% of this population has a degree of pain at one year post FDR, 11% are in moderate to very severe pain. 95% of this population has a degree of disability one year post injury, with 16% being moderately to very severely disabled. Patients in moderate to very severe pain were more likely to require medication. Patients who were moderately to severely disabled were more likely to be older and non working.

It is surprising that such a large proportion of people are left with a degree of pain one year post fracture, with 11% of people being left with a degree of pain that could interfere with their life. Interestingly the percentage of subjects who have no pain (37%) is much greater than those who have no disability (5%). This would suggest that disability is a greater problem to these patients than pain.

The pain prevalence for subjects in this study very closely matched that of MacDermid et al (2003) [8]. In their cohort study of 129 patients following FDR over one year, 11% of their sample had moderate to very severe pain one year post FDR and 32% had no pain. This suggests that levels of pain may be stable across different populations.

The VAS has been criticised as a measure of pain as it requires ability to understand the abstract concept of the VAS line and then relate it to distance from a zero mark. It also requires the use of a paper and pen which is of particular importance in the present study as 50% had injured their dominant hand [11]. However, it has been shown to be a valid and reliable measurement of pain and has been agreed upon by the International Association for the Study of Pain as an appropriate measure of pain for clinical trials [10,12].

Despite the potential influence of responder bias and inadequacies of the VAS as an outcome measure it is fair to conclude that this study highlights a small but important number of patients suffering moderate to very severe pain one year following a FDR. However, it is important to note that this study only takes a snap shot at one year post injury and if the participants had been followed for a longer period of time, further recovery may have occurred.

Nearly all patients reported some degree of disability, with 16% reporting moderate to very severe disability. It is possible that mild disability is a normal finding within this population and not related to the injury they have received. The only other study to investigate level of disability one year post fracture is MacDermid et al (2003), in their study only 46% of the sample presented with disability at one year post FDR and only 7% had moderate to very severe disability [8]. This discrepancy may be due to the present study being influenced by responder bias as only 35% of patients contacted responded. Also MacDermid et al (2003) utilised the Patient Rated Wrist Evaluation (PRWE) which may not be as sensitive to disability as the DASH and, hence the level of disability may have been underestimated in their sample [8,9,13,14]. The DASH has been shown to be one of the most valid and reliable measures of disability in the wrist and other joints of the upper extremity [15].

The DASH is applicable to all regions of the upper limb adding to its strength as a suitable measure. The results can be comparable to other studies of a similar nature involving other regions of the upper limb [16]. This gives the DASH an advantage over other wrist disability measures such as the patient rated wrist evaluation (PRWE) which only measures disability of the wrist, meaning that future research into disability prevalence of other regions of the upper limb could not be compared[8]. However, as the DASH concentrates on the disability of the upper limb it can be criticised for its lack of pain measurement hence the need for a visual analogue scale (VAS) alongside the DASH[14].

Despite the potential problems with responder bias the present study demonstrates that there are a small but important group of patients (16% of this sample) who are suffering moderate to very severe disability one year post injury. Similar distributions were seen when the data was analysed separately for males and females.

The present study showed that the main features that were significantly associated with moderate to very severe pain were the need for pain medication, probably as a consequence of poor outcome. The features associated with disability were being over the age of 65 years, working status and need for pain medication. Working status may be a confounder for age as older people are less likely to work.

The results of this study differ from MacDermid et al (2002) who also explored the associations between demographics and pain and disability in this patient group [17]. In their research high levels of pain and disability were associated with claims for compensation, low education levels and radial shortening (a side effect of FDR), but they found no association with age. The disparity of the results between these two studies may be because the proportion of patients in the present study claiming compensation was only 7% (n = 7) compared to 14% (n = 17) in the MacDermid et al (2002) study. The present study did not explore patient's education level or any long term physical side effect of the injury (such as radial shortening). Radial shortening is another potential confounder associated with pain and disability [8]. This study was unable to make associations between these due to inconsistencies in the medical documentation.

Smoking status has been found to be an association with musculoskeletal pain [18]. The present study failed to show any association between smoking status and pain or disability. This may well be due to the small numbers of smokers within the present study in comparison to the Palmer et al (2003) study [18].

The response rate for completed questionnaires was 35% and there were significant differences between the responders and non-responders in terms of both sex and age. The low response rate could have lead to several bias' in this study, where the subjects may have only responded if they were actually having a problem with their injury one year post fracture. Some of the subjects may not actually have experienced fractures and 25% of the original sample was aged over 75 years of age. Questionnaires place a burden on vision, dexterity, memory and literacy [19]. This may have meant that the elderly subjects had difficulty completing the questionnaire and were, therefore under-represented in this study.

On admission to A & E the patient is assigned a diagnosis code which is entered onto the Emergency Department Information System (EDIS). There is potential for error here as the patient is usually given a preliminary diagnosis in A & E, the formal diagnosis is then given at fracture clinic when the X-ray films have been reviewed by the consultant. 6% of patients who responded to the questionnaire were diagnosed as a FDR in A & E then were assigned a different diagnosis in fracture clinic. This means it is also possible that some patients who do have a FDR may have been entered into the system as having soft tissue injuries when in fact they actually have fractures. These patients will not have been located in this study as they will not have been picked up by the EDIS search. This means that the total population of FDR in this area, during that time period may not have been included in the study.

Examination of the medical records showed that different diagnostic descriptions were used for the radiographic films. Not all diagnosis complied with the AO classification system of intra and extra articular fracture classification as recommended by McRae and Esser (2002) [20]. Some medical records did mention the presence or absence of an articular fracture but this was not consistent among all records. This is in keeping with research by Kreder et al (1996) which asked varying different clinicians to view 30 films of fracture of the distal radius [21]. They also found differing levels of consistency in the diagnosis of the films. The importance of this discrepancy for patient management is that the type of fracture can determine the prognosis, as intra-capsular fractures are deemed to be more likely to have long term influences on the patient's recovery [22]. The incomplete documentation also negated any further analysis of subgroups of patients within this study.

A further limitation of the present study was that it was only based on one area of the UK and therefore, it is difficult to make comparisons or generalisations to other areas of the UK. Further research could focus on surveying other regions of the UK to look for trends in prevalence.

## Conclusion

This research shows that a proportion of patients, from this sample, are suffering moderate to very severe pain (11%) and moderate to very severe disability (16%) one year after their injury. Older age, non-working status and pain medication usage are all associated with, or a consequence of, poor outcome one year after injury. Pain and disability are a significant issue for these patients highlighting the public health implications and the need for further study of appropriate management strategies. This is of particular importance as these patients are primarily elderly females who will be requiring some form of social assistance, whether that is from the state or as a burden on family and friends.

## Competing interests

The authors declare that they have no competing interests.

## Authors' contributions

CM carried out the design, ethical approval, methodology, data collection, statistical analysis and draft of the manuscript. JLB participated in the design of the study and the statistical analysis. All authors read and approved the final manuscript.

## Pre-publication history

The pre-publication history for this paper can be accessed here:



## Supplementary Material

Additional file 1 **DASH questionnaire.** Questionnaire sent to patients Click here for file

Additional file 2 **VAS.** Questionnaire sent to patients Click here for file
