# Supplementary material for: The prevalence of pain and disability one year post fracture of the distal radius in a UK population: A cross sectional survey
Source: BMC Musculoskelet Disord. 2008 Sep 29;9:129. doi: 10.1186/1471-2474-9-129 (PMC2576249; doi:10.1186/1471-2474-9-129)
Supplement: Additional file 1 — DASH questionnaire. Questionnaire sent to patients [file 1471-2474-9-129-S1.pdf]

THE DASH

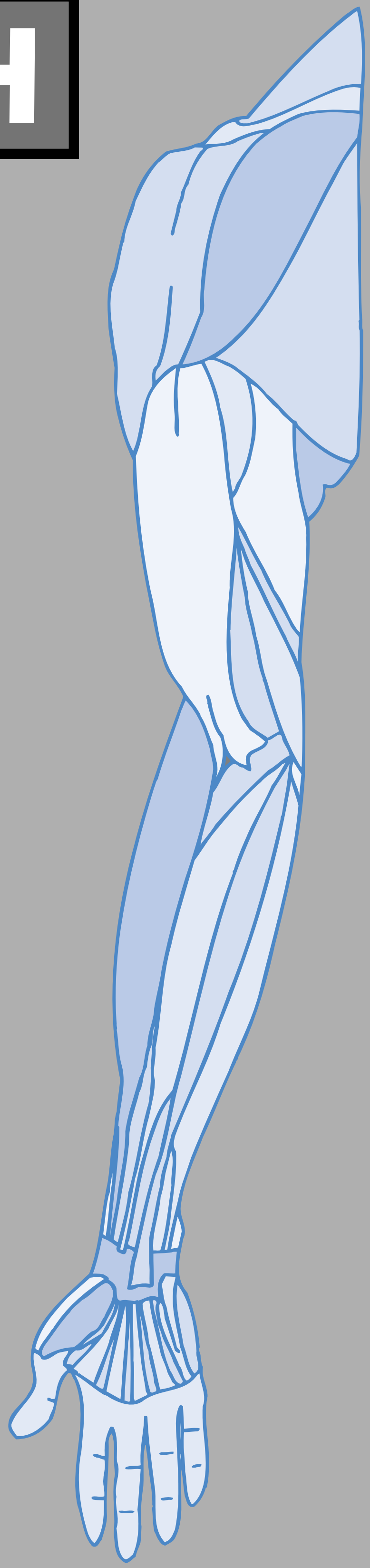

INSTRUCTIONS

This questionnaire asks about your symptoms as well as your ability to perform certain activities.

Please answer *every question*, based on your condition in the last week, by circling the appropriate number.

If you did not have the opportunity to perform an activity in the past week, please make your *best estimate* on which response would be the most accurate.

It doesn't matter which hand or arm you use to perform the activity; please answer based on your ability regardless of how you perform the task.

Disabilities of the Arm, Shoulder and Hand

Please rate your ability to do the following activities in the last week by circling the number below the appropriate response.

	NO DIFFICULTY	MILD DIFFICULTY	MODERATE DIFFICULTY	SEVERE DIFFICULTY	UNABLE
1. Open a tight or new jar.	1	2	3	4	5
2. Write.	1	2	3	4	5
3. Turn a key.	1	2	3	4	5
4. Prepare a meal.	1	2	3	4	5
5. Push open a heavy door.	1	2	3	4	5
6. Place an object on a shelf above your head.	1	2	3	4	5
7. Do heavy household chores (e.g., wash walls, wash floors).	1	2	3	4	5
8. Garden or do yard work.	1	2	3	4	5
9. Make a bed.	1	2	3	4	5
10. Carry a shopping bag or briefcase.	1	2	3	4	5
11. Carry a heavy object (over 10 lbs).	1	2	3	4	5
12. Change a lightbulb overhead.	1	2	3	4	5
13. Wash or blow dry your hair.	1	2	3	4	5
14. Wash your back.	1	2	3	4	5
15. Put on a pullover sweater.	1	2	3	4	5
16. Use a knife to cut food.	1	2	3	4	5
17. Recreational activities which require little effort (e.g., cardplaying, knitting, etc.).	1	2	3	4	5
18. Recreational activities in which you take some force or impact through your arm, shoulder or hand (e.g., golf, hammering, tennis, etc.).	1	2	3	4	5
19. Recreational activities in which you move your arm freely (e.g., playing frisbee, badminton, etc.).	1	2	3	4	5
20. Manage transportation needs (getting from one place to another).	1	2	3	4	5
21. Sexual activities.	1	2	3	4	5

DISABILITIES OF THE ARM, SHOULDER AND HAND

	NOT AT ALL	SLIGHTLY	MODERATELY	QUITE A BIT	EXTREMELY
22. During the past week, *to what extent* has your arm, shoulder or hand problem interfered with your normal social activities with family, friends, neighbours or groups? *(circle number)*	1	2	3	4	5

	NOT LIMITED AT ALL	SLIGHTLY LIMITED	MODERATELY LIMITED	VERY LIMITED	UNABLE
23. During the past week, were you limited in your work or other regular daily activities as a result of your arm, shoulder or hand problem? *(circle number)*	1	2	3	4	5

Please rate the severity of the following symptoms in the last week. *(circle number)*

	NONE	MILD	MODERATE	SEVERE	EXTREME
24. Arm, shoulder or hand pain.	1	2	3	4	5
25. Arm, shoulder or hand pain when you performed any specific activity.	1	2	3	4	5
26. Tingling (pins and needles) in your arm, shoulder or hand.	1	2	3	4	5
27. Weakness in your arm, shoulder or hand.	1	2	3	4	5
28. Stiffness in your arm, shoulder or hand.	1	2	3	4	5

	NO DIFFICULTY	MILD DIFFICULTY	MODERATE DIFFICULTY	SEVERE DIFFICULTY	SO MUCH DIFFICULTY THAT I CAN'T SLEEP
29. During the past week, how much difficulty have you had sleeping because of the pain in your arm, shoulder or hand? *(circle number)*	1	2	3	4	5

	STRONGLY DISAGREE	DISAGREE	NEITHER AGREE NOR DISAGREE	AGREE	STRONGLY AGREE
30. I feel less capable, less confident or less useful because of my arm, shoulder or hand problem. *(circle number)*	1	2	3	4	5

DASH DISABILITY/SYMPTOM SCORE = $\left[\frac{\text{sum of n responses}}{n} - 1\right] \times 25$, where n is equal to the number of completed responses.

A DASH score may <u>not</u> be calculated if there are greater than 3 missing items.

WORK MODULE (OPTIONAL)

The following questions ask about the impact of your arm, shoulder or hand problem on your ability to work (including homemaking if that is your main work role).

Please indicate what your job/work is:____

❐ I do not work. (You may skip this section.)

Please circle the number that best describes your physical ability in the past week. Did you have any difficulty:

	NO DIFFICULTY	MILD DIFFICULTY	MODERATE DIFFICULTY	SEVERE DIFFICULTY	UNABLE
1. using your usual technique for your work?	1	2	3	4	5
2. doing your usual work because of arm, shoulder or hand pain?	1	2	3	4	5
3. doing your work as well as you would like?	1	2	3	4	5
4. spending your usual amount of time doing your work?	1	2	3	4	5

SPORTS/PERFORMING ARTS MODULE (OPTIONAL)

The following questions relate to the impact of your arm, shoulder or hand problem on playing *your musical instrument or sport or both*.
If you play more than one sport or instrument (or play both), please answer with respect to that activity which is most important to you.

Please indicate the sport or instrument which is most important to you:______________________________

❑ I do not play a sport or an instrument. (You may skip this section.)

Please circle the number that best describes your physical ability in the past week. Did you have any difficulty:

	NO DIFFICULTY	MILD DIFFICULTY	MODERATE DIFFICULTY	SEVERE DIFFICULTY	UNABLE
1. using your usual technique for playing your instrument or sport?	1	2	3	4	5
2. playing your musical instrument or sport because of arm, shoulder or hand pain?	1	2	3	4	5
3. playing your musical instrument or sport as well as you would like?	1	2	3	4	5
4. spending your usual amount of time practising or playing your instrument or sport?	1	2	3	4	5

SCORING THE OPTIONAL MODULES: Add up assigned values for each response; divide by 4 (number of items); subtract 1; multiply by 25.
An optional module score may not be calculated if there are any missing items.

INSTITUTE FOR WORK & HEALTH
INSTITUT DE RECHERCHE SUR LE TRAVAIL ET LA SANTÉ

©IWH & AAOS & COMSS 1997
