# Supplementary material for: The prevalence of pain and disability one year post fracture of the distal radius in a UK population: A cross sectional survey
Source: BMC Musculoskelet Disord. 2008 Sep 29;9:129. doi: 10.1186/1471-2474-9-129 (PMC2576249; doi:10.1186/1471-2474-9-129)
Supplement: Additional file 2 — VAS. Questionnaire sent to patients [file 1471-2474-9-129-S2.pdf]

QUADRUPLE VISUAL ANALOGUE SCALE

Patient ID Number:______________________________

Please read carefully:

Instructions: Please circle the number that best describes the question being asked.

Note: If you have more than one complaint, please answer each question for each individual complaint and indicate the score for each complaint. Please indicate your pain level right now, average pain, and pain at its best and worst.

Example:

Elbow (2) — Wrist (5) — Shoulder (8)

No pain ______________________________ **worst possible pain**

0 1 (2) 3 4 (5) 6 7 (8) 9 10

---

1 – What is your pain RIGHT NOW?

No pain ______________________________ **worst possible pain**

0 1 2 3 4 5 6 7 8 9 10

2 – What is your TYPICAL or AVERAGE pain?

No pain ______________________________ **worst possible pain**

0 1 2 3 4 5 6 7 8 9 10

3 – What is your pain level AT ITS BEST (How close to "0" does your pain get at its best)?

No pain ______________________________ **worst possible pain**

0 1 2 3 4 5 6 7 8 9 10

4 – What is your pain level AT ITS WORST (How close to "10" does your pain get at its worst)?

No pain ______________________________ **worst possible pain**

0 1 2 3 4 5 6 7 8 9 10

OTHER COMMENTS:

____

____

____
Examiner

Reprinted from *Spine*, 18, Von Korff M, Deyo RA, Cherkin D, Barlow SF, Back pain in primary care: Outcomes at 1 year, 855-862, 1993, with permission from Elsevier Science.
